# Community‐led monitoring of HIV and viral hepatitis services: lessons learned and impacts from India and Indonesia

**DOI:** 10.1002/jia2.26373

**Published:** 2024-10-03

**Authors:** Giten Khwairakpam, Rajkumar Nalinikanta, Caroline Thomas, Solange L. Baptiste, Elise Lankiewicz

**Affiliations:** ^1^ TREAT Asia/amfAR Bangkok Thailand; ^2^ Community Network for Empowerment (CoNE) Manipur India; ^3^ Yayasan Peduli Hati Jakarta Indonesia; ^4^ International Treatment Preparedness Coalition ‐ Global (ITPC) Johannesburg South Africa; ^5^ Andelson Office of Public Policy/amfAR Washington DC USA

1

Community‐led monitoring (CLM) assesses healthcare services and improves health outcomes. It provides insights about the state of local or national HIV responses, assisting managers and policymakers to improve services under the framework of availability, accessibility, acceptability and quality (AAAQ) [1]. Interest in CLM is growing, with support from the International AIDS Society, the Joint United Nations Programme on HIV/AIDS (UNAIDS), the Global Fund to Fight AIDS, TB, and Malaria, and the United States President's Emergency Plan for AIDS Relief (PEPFAR) [2, 3, 4, 5].

The Community‐led Monitoring Project in Asia [6] was initiated in 2021 by the Community Network for Empowerment (CoNE) in Manipur, India, Yayasan Peduli Hati Bangsa in Indonesia, the International Treatment Preparedness Coalition—Global (ITPC) and amfAR's TREAT Asia programme. CoNE and Peduli Hati are monitoring 12 health facilities from local districts to referral hospitals that provide public services for HIV and viral hepatitis. CLM indicators are based on national guidelines and policies and target essential components of the AAAQ framework covering HIV, hepatitis B (HBV) and hepatitis C (HCV).

**Table 1:** Monitoring framework

Main issue/problem	Problem description/focus	Quantitative indicator	Quantitative indicator description (includes disaggregation)	Indicator type	Data collection source	Qualitative questions (open‐ended)	Population/group to respond to qualitative questions
Access to preferred first line	Transition of dolutegravir‐based first‐line ARV	Guidelines/regulation drafted or published	Output indicator	Custom	MOH dept leading the guidelines development		
Insufficient testing/treatment services	Decentralize HIV testing and treatment services & to primary care and enable all primary services to provide HIV testing and access to ARV treatment	Number of decentralized HIV testing services (baseline vs. November 2021)	Number of districts with HIV test services (compared to the total number of all districts in Indonesia, *n* = 514)	Same as NSP	Quarterly report from MOH	Quality of services? Healthcare providers to validate or not information reported by the Ministry/on availability of services/and recipient of care (about their experience)	1. All key population and women of general population and youth 2. Recipient of care 3. Healthcare providers
		Number of decentralized HIV treatment services (baseline vs. November 2021)	Number of districts with HIV treatment services (compared to the total number of all districts in Indonesia, *n* = 514)	Same as NSP	Quarterly report from MOH	Quality of services? Healthcare providers to validate or not information reported by the Ministry/on availability of services/and recipient of care (about their experience)	1. All key population and women of general population and youth 2. Healthcare providers 3. Healthcare providers
Concentration only in priority provinces	Expanding access to integration of services (viral load monitoring, EID/early infant diagnosis and TB)	Number of rapid molecular machines procured and used for viral load monitoring, EID and TB (baseline vs. November 2021)	Govt target for procurement of machines? Where are they planned to be distributed?	Same as NSP	Quarterly report from MOH—check by province and the national distributor		
		Number of VL tests administered	Disaggregation by site: provinces, city/district/health facility	Same as NSP	National data: quarterly report	Why people are not getting a VL test? Cost? Availability?	1. Recipient of care 2. Healthcare providers
		Number of PLHIV who have received a viral load test result	Disaggregation by site: provinces, city/district/health facility	Same as NSP	National data: quarterly report	Why people are not getting the VL test result? Checking with the healthcare provider	1. Recipient of care 2. Healthcare providers

In April 2023, the project transitioned to a digital platform with the support of amfAR's Andelson Office of Public Policy. It is one of the first CLM projects in the Asia‐Pacific region to make real‐time data publicly available through an online dashboard and is the only global CLM project monitoring HBV and HCV indicators. The implementation of the project involved components across four phases:

1. ***Development of a monitoring framework***: In the first phase, community members, who are people living with HIV or who use drugs and are associated with CoNE or Peduli Hati, discussed problems in accessing services and prioritized what they wanted to monitor through a monitoring framework (Table 1). The monitoring framework could be adapted for other health issues. Qualitative and quantitative indicators were further developed in the form of survey tools [7].
2. ***Digitalization***: Digitalizing data collection and reporting processes facilitated engagement with care recipients and real‐time availability of data. The CommCare application was chosen due to its ease of use, data security, possibility of offline data collection, and adaptability for use with tablets and mobile phones. Pilot testing of the tools through mock data collection was conducted and required changes were made. Data enumerators, who are peers from the community members and not health facility staff, were trained on the data collection and submission processes using the digitalized platform.

A data dashboard was developed that synchronizes uploaded data in real‐time [8, 9]. The dashboard generates data visualizations through graphs and figures. This makes it easier to communicate findings to facility managers and decision‐makers to support advocacy around the quality of care reported by the care recipients.

3 ***Monitoring***: The data enumerators interviewed care recipients for responses on the indicators using tablets or mobile phones. Interviews took about 30 minutes and were conducted both at facilities and at locations where community members frequently visits, such as coffee shops. Respondents were a convenience sample and not compensated. Internet connectivity was not required during the process as the software stores data upon submission and synchronizes with the dashboard as soon the data enumerator had internet access. The data collection process not only recorded user responses to questions about the care they were receiving but also served as an opportunity to hear about people's inability to access services, concerns about social justice or human rights violations. The community partners were able to rapidly initiate advocacy efforts to address shortcomings that needed immediate attention. Efforts to address some of the broader social justice or human rights issues commenced immediately or soon after the data collection process [10].
4 ***Advocacy***: Insights from monitoring are utilized to drive adjustments in facility operations and broader policy decisions, and vice versa. Meetings with facility managers and decision‐makers were organized quarterly, or as and when needed, to communicate findings and co‐create solutions to the issues. Reports focused on individual health facilities can be generated from the dashboard and shared with the facility managers to help them understand how care recipients perceive the services.

In 2022, paper‐based data collection required data enumerators to record responses on paper and later manually input the same responses into a database for analysis. Digitalization allowed enumerators to directly record responses into tablets during data collection, with data visualized automatically on the dashboard in near real‐time. This reduced the time required for data entry, made data available in real‐time for advocacy and lowered opportunities for data entry errors given the single submission. From April to December 2023, the project reached out to 1593 care recipients in comparison to the 120 people reached through interviews and focus groups using paper forms between January and December in 2022. Among those participating in the digitalized CLM process, 76% were 25–49 years of age, 71% were male, 28% were female and 0.56% were transgender. Respondents were receiving care for HIV (62%), HBV (3%) and HCV (34%). During this timeframe, the project has led to the improvement of care services and facilitated advocacy around human rights and social justice, as described in the examples below:

- Successfully resolved seven occurrences of shortages and stock‐outs of HIV and HCV medicines. CLM teams immediately responded to stockouts captured by enumerators by alerting provincial authorities for rapid resolution.
- Data enumerators advocated for various strategies to improve access to services. Seven people living with HIV in India were transferred to care facilities they identified to be more convenient for them, demonstrating CLM's role in filling care coordination gaps where facility staff may be overstretched. In Indonesia, they facilitated home delivery of Antiretroviral treatment for a visually challenged person. The two project partners linked 174 people living with HIV to HCV testing and HIV viral load testing, 19 people to HBV treatment, 37 to HCV treatment and 42 people to HCV Sustained Virological Resposne 12 test to know if they have been cured of the infection.
- In India, 20 children living with or affected by HIV were linked to social security schemes providing financial support for their education. Programme staff encountered two students expelled from hostels because of their HIV status. With advocacy initiatives to the state HIV Ombudsman, they were readmitted to the hostel. Three women living with HIV who were denied their rights were linked to free legal assistance.

CLM activities are an effective process to bring about positive changes in healthcare services. For CLM to deliver optimal impact, we recommend:

- Use of monitoring frameworks that prioritize essential indicators to minimize burdens on data enumerators.
- Investment in digitalization of tools and an online dashboard to facilitate real‐time data visualization to optimize use in advocacy.
- Support for data enumerators with mobile internet data airtime, travel during data collection, remuneration for their time and training to understand packages of services in different national programmes to optimize linkages.
- Use of CLM platforms to address broader challenges beyond treatment access and uptake to identify human rights violations and facilitate redress.

CLM is a mechanism where care recipients’ perceptions of the essential components of healthcare are captured and leveraged to advocate for changes in service delivery. Our observations indicate that CLM can also play a critical role in addressing urgent individual‐level human rights and care access issues in a more rapid timeframe through effective co‐problem‐solving and advocacy.

## COMPETING INTERESTS

The project is funded by ViiV Healthcare. The authors otherwise have no competing interests to declare.

## AUTHORS’ CONTRIBUTIONS

GK led the drafting and writing of the manuscript. EL reviewed the draft and final manuscript. RN, CT and SLB provided inputs and edits. All authors approved the final version of the manuscript.

## FUNDING

The CLM in Asia project is supported by ViiV Healthcare and amfAR.

## Data Availability

Data sharing is not applicable to this article as no datasets were generated or analysed during the current study.
